# Author Correction: Optical manipulation of Rashba-split 2-dimensional electron gas

**DOI:** 10.1038/s41467-022-32345-6

**Published:** 2022-08-03

**Authors:** M. Michiardi, F. Boschini, H.-H. Kung, M. X. Na, S. K. Y. Dufresne, A. Currie, G. Levy, S. Zhdanovich, A. K. Mills, D. J. Jones, J. L. Mi, B. B. Iversen, Ph. Hofmann, A. Damascelli

**Affiliations:** 1 Quantum Matter Institute, University of British Columbia, Vancouver, BC V6T 1Z4 Canada; 2 Department of Physics & Astronomy, University of British Columbia, Vancouver, BC V6T 1Z1 Canada; 3 Max Planck Institute for Chemical Physics of Solids, Dresden, Germany; 4 Centre Énergie Matériaux Télécommunications, Institut National de la Recherche Scientifique, Varennes, QC J3X 1S2 Canada; 5 Department of Chemistry, Aarhus University, 8000 Aarhus C, Denmark; 6 Department of Physics and Astronomy, Interdisciplinary Nanoscience Center, Aarhus University, 8000 Aarhus C, Denmark

**Keywords:** Electronic properties and materials, Spintronics

Correction to: *Nature Communications* 10.1038/s41467-022-30742-5, published online 02 June 2022

The original version of this article acknowledged funding from the ‘The Department of National Defense (DND)’. This has been corrected to ‘the Department of National Defence’. This error has now been corrected in both the PDF and HTML versions of the article.

