# Supplementary material for: Optical manipulation of Rashba-split 2-dimensional electron gas
Source: Nat Commun. 2022 Jun 2;13:3096. doi: 10.1038/s41467-022-30742-5 (PMC9163084; doi:10.1038/s41467-022-30742-5)
Supplement: Supplementary file 1 — Supplemetary Information [file 41467_2022_30742_MOESM1_ESM.pdf]

Supplemetary Information for "Optical manipulation of Rashba-split 2-Dimensional Electron Gas"

M. Michiardi,[1,2,3,*] F. Boschini,[1,2,4] H.-H. Kung,[1,2] M. X. Na,[1,2]
S. K. Y. Dufresne,[1,2] A. Currie,[1,2] G. Levy,[1,2] S. Zhdanovich,[1,2] A. K. Mills,[1,2]
D. J. Jones,[1,2] J. L. Mi,[5] B. B. Iversen,[5] Ph. Hofmann,[6] and A. Damascelli[1,2,†]

[1]*Quantum Matter Institute, University of British Columbia, Vancouver, BC V6T 1Z4, Canada*

[2]*Department of Physics & Astronomy, University of British Columbia, Vancouver, BC V6T 1Z1, Canada*

[3]*Max Planck Institute for Chemical Physics of Solids, Dresden, Germany*

[4]*Centre Énergie Matériaux Télécommunications, Institut National de la Recherche Scientifique, Varennes, Québec J3X 1S2, Canada*

[5]*Department of Chemistry, Aarhus University, 8000 Aarhus C, Denmark*

[6]*Department of Physics and Astronomy, Interdisciplinary Nanoscience Center, Aarhus University, 8000 Aarhus C, Denmark*

I. EFFECT OF PHOTOVOLTAGE ON THE PHOTOEMISSION EXPERIMENT

In the presence of a band bending potential, free carriers generated by an optical excitation are swept apart by the electric field ($\boldsymbol{E_{SCR}}$) in the space-charge region. The dissociated carriers generate a field ($\boldsymbol{E_{PV}}$) in the opposite direction of $\boldsymbol{E_{SCR}}$, producing the photovoltage (PV) effect. The PV not only alters the electrostatic environment of the electrons inside the material (i.e. softening the band bending) but also has repercussions on the the photoemission process itself. These effects have been the subject of several dedicated studies [1–3]; here we highlight the phenomenological implications and the corresponding data treatment process.

The sign of $\boldsymbol{E_{PV}}$ depends on the equilibrium band bending of the material. For a downward band bending – as it is the case in our experiment – $\boldsymbol{E_{PV}}$ in the vacuum region points toward the surface, resulting in an outward accelerating force $\boldsymbol{F_{PV}}$ acting on the negative charges (electrons) outside the material. A schematic of this effect is presented in Fig. S1**a**, showcasing the effect of the photovoltage on the time-resolved photoemission process at positive and negative time delays.

In the left panel of Fig. S1**a** (positive delays), the pump pulse comes first, establishing the photovoltaic field in the area of the sample illuminated. Subsequently, the probe pulse emits an electron from the pumped area, and its kinetic energy is increased by the PV field. The kinetic energy of the photo-electron at positive delays is a reflection of its binding energy inside the material as well as the magnitude of the PV. For a simple surface state, this manifests as a rigid shift of the photoemission spectrum with respect to equilibrium, as shown in Fig. S1 **b** where we compare the ARPES spectrum with and without the presence of the pump.

The situation is slightly more complex at negative delays. The electron is photoemitted first by the probe pulse; when the pump pulse impinges on the sample after some delay Δt, the outgoing photoelectron at the distance $v\Delta t$ from the sample, where v is its velocity, is pushed by $\boldsymbol{F_{PV}}$. The smaller the delay, the stronger is the force felt, resulting in a gradual increase in the kinetic energy of the electrons at negative delays up to the time zero. These effects are clearly observed in time resolved spectra taken over long timescales (hundreds of picoseconds), see Fig. S1**c,d**.

* mmichiardi@phas.ubc.ca
† damascelli@physics.ubc.ca

In order to provide a consistent picture of the electronic dispersion in the material and to extract information of electron transport properties, the data in the main text are plotted against the non-equilibrium electron quasi-Fermi level (E_{Fn}). To extract the E_{Fn}, we fit the energy distribution curve (EDC) integrated around the Fermi wave-vector of the topological surface state (red area in Fig. S1**b**) with a Fermi-Dirac distribution function. This is possible because the momentum-integrated spectral function of a linearly dispersing band corresponds to its density of states [4, 5], and it allows us to reliably obtain the location of the non-equilibrium chemical potential of the sample at each time delay. Figure. S1**d** displays the time-resolved trace around the topological surface state (TSS) Fermi wave-vector and the extracted E_{Fn} (red markers). The time trace obtained around the Brillouin zone (BZ) center (blue area in Fig. S1**b**) shown in panel **c** is then re-scaled by subtracting the E_{Fn} energy at each delay; the resulting plot is shown in Fig. S1**e**. This methods removes artifacts in the photoemitted electron kinetic energy and provides a clear picture of the non-equilibrium "binding energy" of the electrons inside the material. As we can see in the corrected map of Fig. S1**e**, the system is in equilibrium at negative delays – i.e. the quantum well states' (QWS) energy does not change –, while at positive delays the impact of the PV effect on the band-bending is clearly discernible.

II. LIFETIME OF THE PHOTOVOLTAGE EFFECT ON 2DEGS

We discussed the effect of the PV on the energy and spin splitting of the quantum well states (QWSs) in the main text. The lifetime of the PV can be extracted from the time-resolved trace at BZ center in Fig.S1**e**, by tracking the time needed for the QWSs to return to their equilibrium energy. The QWS1 and QWS2 minima are obtained by fitting the EDCs for each pump-probe delay with a phenomenological model consisting of a single (double) Lorentzian profile for negative (positive) delays, multiplied by the Fermi distribution function. Subsequently, the evolution of the energy minima for both QWSs, are fitted using a step function at time zero and an exponential decay for positive delays, convoluted by the temporal resolution. The step function captures the sudden change in the electrostatic environment due to the onset of the PV, while the exponential function captures the return to equilibrium condition due to diffusion of electrons and holes from the illuminated area to the surrounding region. We extract the lifetime of the photovoltaic

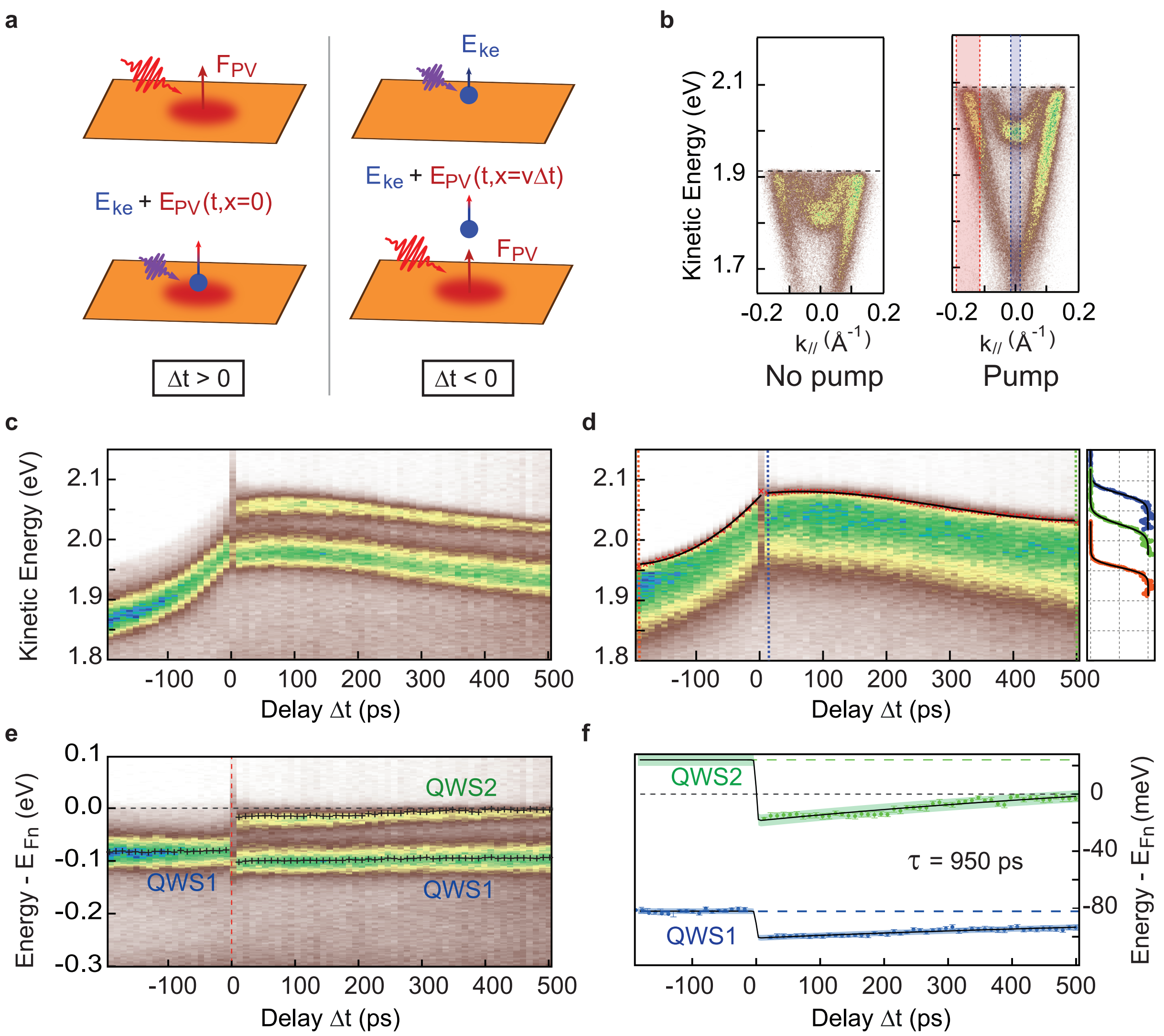

FIG. S1. **Effect of photovoltage (PV) on the photoemission spectra.** **a**: Schematics of the time-resolved photoemission process in a semiconductor with downward band bending and pump-induced PV. For $\Delta t > 0$ the pump generates a PV and the delayed probe emits an electron whose kinetic energy reflects the change in binding energy as well the magnitude of the photovoltage; for a topological surface state (TSS) electron the kinetic energy, $E_{\rm ke}$, is shifted by $E_{PV} = e \cdot V_{PV}$, where e is the electron charge. For $\Delta t < 0$ the electron is photoemitted before the generation of a PV. After a delay Δt, it receives a kick from the PV field created by the pump pulse; this creates a rigid shift in the $E_{\rm ke}$ of photoemission spectrum, which increases up to the time zero. **b**: ARPES spectra of chemically gated Bi_2Se_3 acquired with the pump beam turned off (left) and on (right, slightly before time zero). The TSS is shifted upwards by E_{PV}. **c,d**: Time-resolved traces of the energy distribution curves (EDC) integrated around the Brillouin zone (BZ) center (**c**) and the TSS Fermi wave-vector (**d**) indicated by blue and red areas in panel **b**, respectively. The non-equilibrium chemical potential at each delay is extracted by fitting EDCs in panel **d** with Fermi-Dirac (FD) distribution functions (black solid lines). Profiles at three characteristic delays (highlighted by vertical dashed lines) are shown in the right panel. **e**: same as in **c**, re-scaled with respect to the quasi-Fermi level. Markers indicate the energy of the QWSs. **f**: The energy minima of QWS1 and QWS2 with respect to E_{Fn} obtained by fitting each EDC in **e** with a Loretzian profile multiplied by a FD distribution. The temporal dynamics are extracted by fitting the QWSs' energy with a step function and a single exponential decay, convoluted with the time resolution. We extract the lifetime of the PV effect to be $950 \pm 50\,$ps.

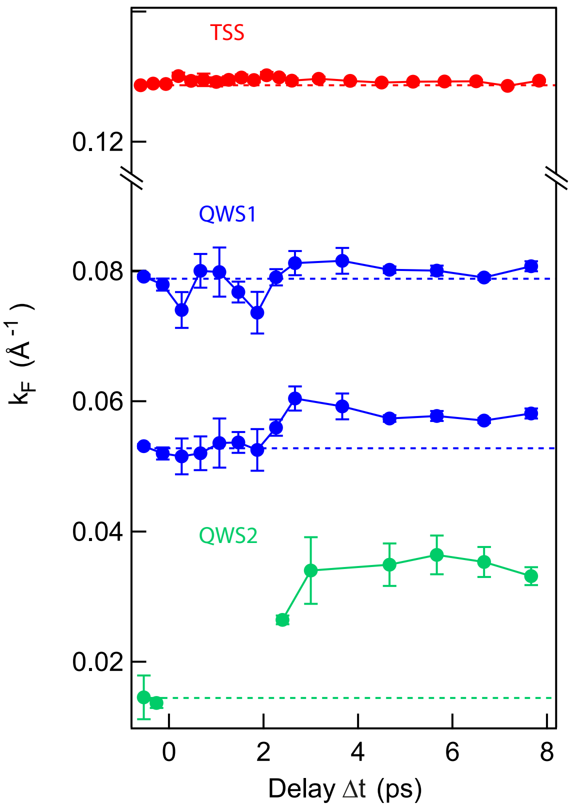

FIG. S2. **Ultrafast temporal evolution of the Fermi wave-vectors.** Wave-vectors at the Fermi level of the Topological state (red), the QWS1 (blue, two branches), the QWS2 (green) as a function of delay time for the datasets presented in Fig. 3 in the main text.

effect to be 950 ps ± 50 ps by fitting QWS1. Since QWS2 is above the chemical potential at negative delays, we impose the decay timescale extracted from QWS1, and find that QWS2 is ≈ 20 meV above the Fermi level at equilibrium. This is consistent with the observation that at negative delays there is no spectral signature of QWS2. We remark that the PV lifetime depends on experimental conditions – such as the spot size of the pump beam – as its value is governed by the diffusion dynamics of carriers from the illuminated area.

III. TEMPORAL DYNAMICS OF K$_F$S AND ELECTRON DENSITY

The photovoltage effect introduces additional electrons on the surface, which causes the QWSs minima to drop to more negative values as detailed in the manuscript and shown in Fig. 3 **b**. The number of additional photo-charges in the system is quantifiable by measuring the Fermi wave-vectors k_F for each band. The $k_F(\Delta t)$ for each QWS and the topological

surface state (TSS) are obtained by fitting the MDC across the Fermi level and are reported in Fig.S2. Both Rashba-split branches are shown for QWS1 in blue, while the splitting is too small for QWS2 to be analyzed within the experimental resolution. The uncertainty is largest between 0 and 2 ps, as the sharpness and intensity of features at E_F is adversely affected by the direct optical excitation and increased electron temperature, respectively. For this reason, extraction of k_F of QWS2 in the 0 – 2 ps time domain is not possible. However, the k_F of both QWSs clearly increase at large positive delays, and the effect is the largest for QWS2 – an expected consequence of the softening of the band bending potential (see Fig.S1 and S4). Using Luttinger's theorem and under the reasonable assumption that the Fermi surfaces are circular, we can use the following formula to extract the electron density per unit cell from the values of k_F :

$$n^e_{UC} = \frac{(\pi k_F^2)}{A^{2D}_{BZ}} n_e \tag{S1}$$

Where A^{2D}_{BZ} is the size of the 2-dimensional Brillouin Zone of Bi_2Se_3 (111) and n_e is the electron degeneracy of the state. The electron density increases by $2.4 \pm 0.5 \cdot 10^{-3}$ (electrons/Unit cell) in QWS2 4 ps after the optical excitation. The value is smaller for QWS1 with $1.3 \pm 0.5 \cdot 10^{-3}$ and it is negligible for the topological surface state.

IV. TEMPORAL EVOLUTION OF THE ELECTRONIC TEMPERATURE

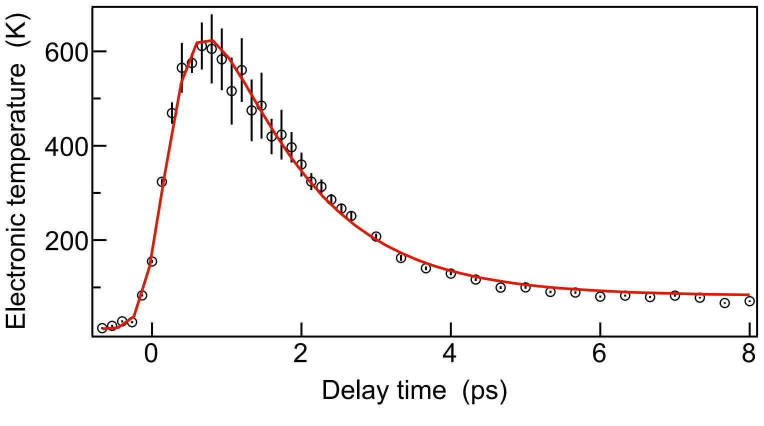

FIG. S3. **Electronic temperature as a function of delay time.** The electronic temperature (open circles) is obtained from the data presented in Fig.3 of the manuscript and is fitted with a two-temperature model (solid red line).

V. STATIC SIMULATIONS OF BAND BENDING AND QUANTUM WELL STATES

To simulate the band bending, we build a one dimensional model along the direction perpendicular to the surface; this is justified under the assumption that the lateral dimensions of interest (here 300 μm, the size of the probe beam) is much larger than the perpendicular one ($\approx$ 50 nm, the width of the space charge region). We employ the modified Thomas-Fermi approximation (MTFA) approach, following the steps described in Ref. 6.

The calculation of the charge densities and electron potential normally requires to solve the coupled Poisson and Schrödinger equations self-consistently, a process that is numerically expensive. However, the MTFA allows to calculate both quantities from the Poisson equation through the introduction of a term, f_{MTFA}, that accounts for the potential barrier at the surface, and reflects the surface confinement-induced quantization of the density of states.

To calculate the band energy as a function of depth x (where $x = 0$ corresponds to the surface), one needs to solve the Poisson equation in one dimension:

$$\frac{d^2V(x)}{dx^2} = -\frac{\rho}{\epsilon} = -\frac{e}{\epsilon_0\epsilon_r}[N_D^+ - N_A^- - n(x) + p(x)], \tag{S2}$$

for the electron potential $V(x)$. The charge ρ in the semiconductor is a function of the concentrations of ionized donor and acceptor atoms in the bulk, N_D^+ and N_A^-, as well as the electron and hole density distributions, $n(x)$ and $p(x)$. These latter values are calculated from the conduction and valence band density of states, $g_{C/V}$, with the introduction of $f_{\mathrm{MTFA}}(x)$ as:

$$n(x) = \int_{E_{\mathrm{CB}}}^{\infty} g_{\mathrm{C}}(E)F(E)f_{\mathrm{MTFA}}(x)dE, \tag{S3}$$

$$p(x) = \int_{-\infty}^{E_{\mathrm{VB}}} g_{\mathrm{V}}(E)[1 - F(E)]f_{\mathrm{MTFA}}(x)dE, \tag{S4}$$

where F(E) is the Fermi-Dirac distribution, and $E_{\mathrm{CB/VB}}$ is the energy of the bottom/top of the conduction/valence band. The term $f_{\mathrm{MTFA}}(x)$ is given by:

$$\begin{aligned} f_{\mathrm{MTFA}}(x) &= 1 - \left[\frac{2x}{L}\left(\frac{E}{k_BT}\right)^{\frac{1}{2}}\left(1 + \frac{E}{E_g}\right)^{\frac{1}{2}}\right], \\ L &= \hbar/(2m^\star k_BT)^{\frac{1}{2}}, \end{aligned} \tag{S5}$$

where E_g is the band gap, k_B is the Boltzmann constant, and T is the temperature.

Parameter	Value	Reference	Description
m_{hl}	0.13 m_e	[8]	light holes effective mass
m_{hh}	0.8 m_e	[8]	heavy holes effective mass
m_e	0.13 m_e	[9]	electron effective mass
ϵ_r	113	[9, 10]	dielectric constant
Eg	0.25 eV	[11, 12]	band gap
N_a	$5{\cdot}10^{18}$ cm^{-3}		density of acceptor ions

TABLE I. Material specific parameters used for simulating the band bending in p-doped Bi_2Se_3. The density of acceptors ions has been chosen in the simulation to correspond to the level of p-doping observed experimentally – where the Fermi level is 20 meV below the valence band maximum.

We solve the MTFA-Poisson equation using the damped Newton numerical method with the boundary conditions: $V(0) = V_0$ and $V(\infty) = 0$. From the calculated $V(x)$, we find the solutions of the Schrödinger equation:

$$-\frac{\hbar^2}{2m^\star}\frac{d^2}{dx^2}\Psi(x) + V(x)\Psi(x) = E\Psi(x), \tag{S6}$$

to obtain the wave-functions and energies of the QWSs. Equation S6 is solved numerically employing the Numerov method. The fully implemented code used in this work to simulate band bending and QWSs has been made freely accessible at Ref. 7.

The material specific parameters we used are summarized in Table I

We set the surface boundary condition $V_0 = -0.54$ eV to simulate the results of the experiment described in Fig. 4 of the main text. This value was extracted by measuring the shift in energy of the TSS Dirac point between a freshly cleaved sample and the chemically gated sample. Thanks to the surface-nature of the TSS, its energy rigidly shifts with the surface potential. The energy profile of $V(x)$ is displayed in Fig. S4**a** with a solid black line. The envelope wave-functions of the QWSs are plotted at their respective energies, revealing the ladder-structure of the QWSs.

The electric field inside the material is obtained from $V(x)$ as $\mathbf{E} = -\frac{dV(x)}{dx}$, and its depth profile is plotted in Fig. S4 **b**. The value of the Rashba spin-orbit coupling constant α, on

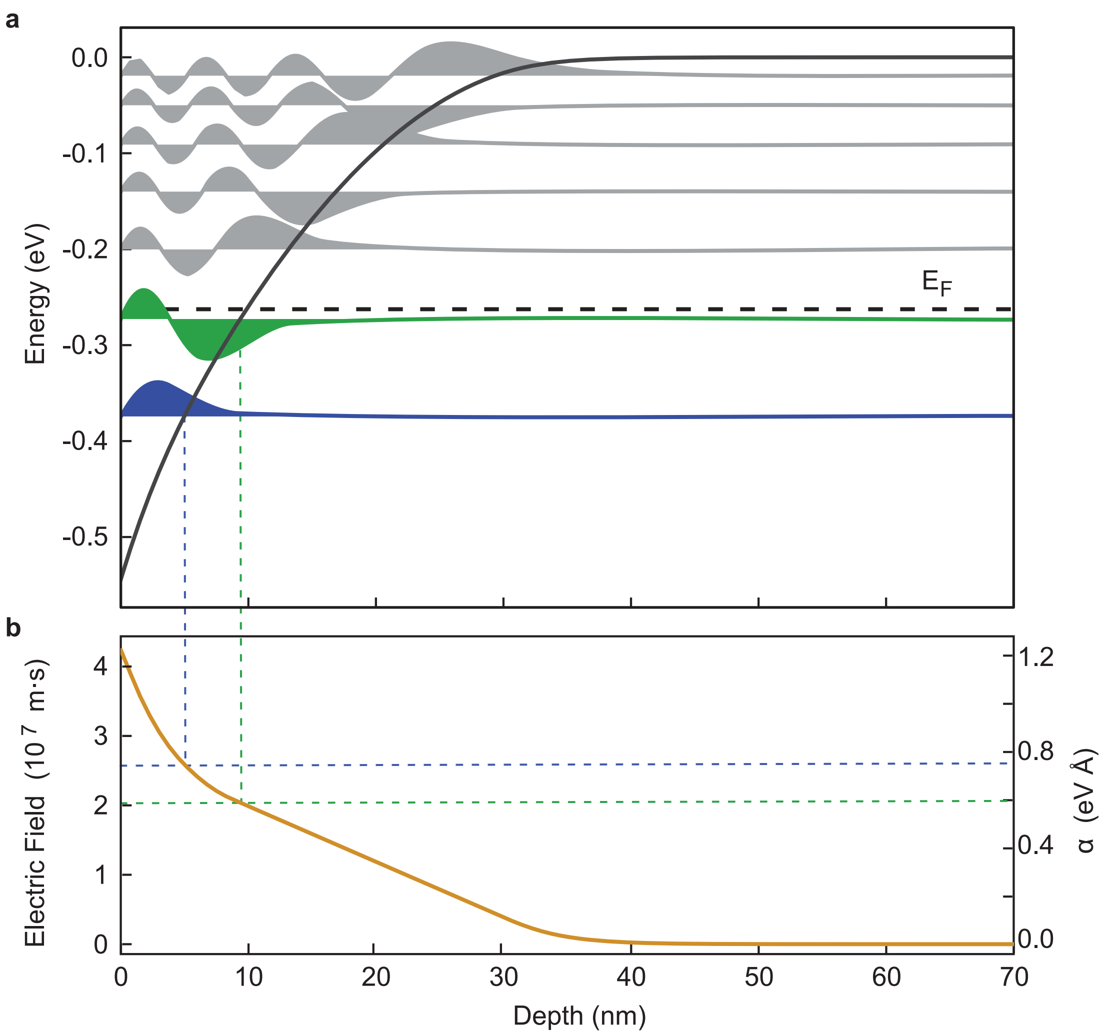

FIG. S4. **Numerical simulation of the band bending and quantum well states.** **a** Band bending profile calculated by solving the MTFA-Poisson equation for the system shown in Fig. 3 of the main text. The calculated quantum well states (QWS) wave-functions are plotted at their respective energies; The first two QWSs are partially occupied. **b** Depth profile of the electric field inside the material and the corresponding Rashba SOC strength α; the field and α for the first two QWSs are indicated by dashed lines. The lowest QWS sits closer to the surface, where the electric field, and likewise the Rashba effect, is stronger.

the right axes, is calculated from the electric field using the equation

$$\alpha = \frac{\hbar^2}{2m_e^\star}\frac{\Delta}{E_g}\frac{2E_g+\Delta}{(E_g+\Delta)(3E_g+2\Delta)}e\mathbf{E}, \tag{S7}$$

where E_g is the material band gap [13]. The spin-orbit energy splitting parameter Δ is here used as a fitting parameter to reproduce the Rashba splitting of QWS1 in equilibrium. The

estimated value of 0.84 eV is later used for all calculations of the Rashba SOC in this system.

VI. DYNAMICAL SIMULATIONS OF BAND BENDING AND QUANTUM WELL STATES IN PUMP-PROBE EXPERIMENTS

To calculate the time-dependent photovoltaic effect on the system under study, one needs to know the spatial distribution of the photo-generated charges, the electron-hole recombination rate, and the carriers diffusion coefficient [14]. In the effort to capture the main findings of our experiments, we work under the following approximations: (1) The diffusion rate is omitted in our calculations; the timescale for the carrier diffusion, extracted from our data, is 950 ps, and has little relevance within the short timescale (< 10 ps) of interest when reproducing the data shown in Fig. 4 in the main text. (2) The electron-hole recombination is taken into account by the introduction of an effective photo-carrier density, $N_{h\nu}$. After photo-carriers are generated, a large percentage recombines in a short time through electron-electron and electron-phonon scattering processes. Only a fraction of photo-generated carriers is swept apart by the electric field without recombining. This percentage of carriers constitute the effective photo-carrier density responsible for the PV effect. (3) Finally, the photo-carrier spatial distribution is rationalized by employing a center of mass approach: the charge distribution is replaced with a single point charge $Q_{n/p} = \mp e \cdot N_{h\nu}$ situated at its center of mass, $\langle x_n \rangle$ and $\langle x_p \rangle$ for electron and holes respectively. The charge carriers' center of mass moves in time within the electric field akin a point charge as:

$$\langle x_{n/p} \rangle (t) = \langle x_{n/p} \rangle (t - \Delta t) \mp \mu E(x) \cdot \Delta t, \tag{S8}$$

where μ is the carrier mobility. Finally, analogously to a capacitor model, one can obtain the time-dependent equation for the PV magnitude [15]:

$$V_{\mathrm{PV}}(t) = \frac{e}{\epsilon_r \epsilon_0} N_{h\nu} [\langle x_n \rangle (t) - \langle x_p \rangle (t)]. \tag{S9}$$

In our simulation, we calculate the band bending from Eq. S2 at each time step using the boundary condition $V_0(t) = V_0(0) + V_{\mathrm{PV}}(t)$. The QWS and Rashba splittings are also calculated at each step through Eqs. S6 and S7. The E_{Fn} at the surface is calculated under the flat quasi-Fermi level approximation [16] by finding the value of E_{Fn} that satisfies charge neutrality:

$$\int_{eV_0}^{E_{Fn}} n(x, E) dE = (Q_0 + e \cdot N_{h\nu}), \tag{S10}$$

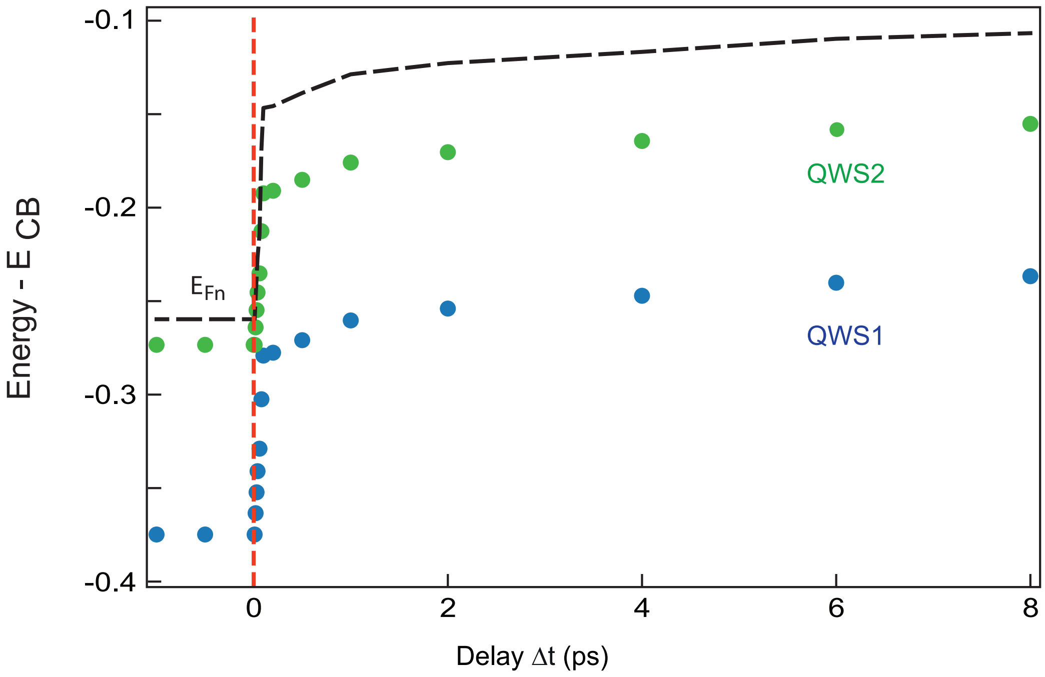

FIG. S5. **Time dependence of the calculated QWSs energies and the electron quasi-Fermi level at the surface.** The energy is scaled with respect to the bulk conduction band energy E_{CB}. At time zero the photovoltage rapidly shifts all the energies: QWS1 and QWS2 are more closely spaced because of the softened band bending. The distance between E_{Fn} and the QWS increases with respect to equilibrium because of the higher electron density at the surface brought by the negative photo-carriers.

where Q_0 is the equilibrium surface charge. For a comparison with our data we use an effective photo-carrier density $N_{h\nu} = 2.5{\cdot}10^{12}\text{cm}^{-2}$, which yields a maximum photovoltage of 180 meV. This value corresponds to the measured shift in the kinetic energy of the TSS Dirac point when the pump impinges on the sample surface.

In Fig. S5, we plot the simulated time dependence of the QWSs minima (blue and green for QWS1 and QWS2, respectively) and of the electron quasi-Fermi level (dashed black line) with respect to the bulk conduction band energy. The PV-induced energy shift at positive delays is larger for QWS1 than for QWS2, as it sits closer to the surface, and the softening of the band bending results in a smaller energy separation between consecutive QWSs. The evolution of E_{Fn} reflects the change of electron density at the surface as direct consequence of the additional photo-generated electrons at the surface. When plotting the QWSs energy

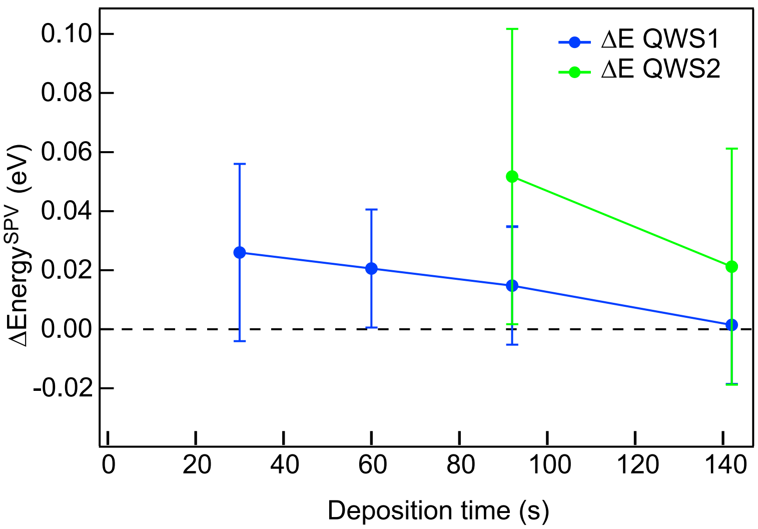

FIG. S6. **Chemical gating dependence of the PV effect on the QWSs.** Photovoltage-induced binding energy shift of the QWSs (blue for QWS1 and green for QWS2) as a function of alkali deposition time on the surface. The energy shift is obtained as the difference between the QWS energy minima at $\Delta t = 20$ and -1 ps.

with respect to E_{Fn}, one obtains the plot of Fig. 4**c** of the main text.

VII. CHEMICAL GATING DEPENDENCE OF THE PHOTOVOLTAGE EFFECT

To elucidate the relation between initial surface doping and the PV effect, we progressively evaporate K on a sample of Bi_2Se_3 and measure the photoemission spectrum before the pump arrival (i.e., at a negative time delay of -1 ps), and 20 ps after the pump arrival. At 20 ps, the system is fully thermalized and bears the signature of the PV effect, namely an increase in binding energy due to the excess electron population at the surface. The change in binding energy, calculated as the difference between the QWS energy minima at $\Delta = 20$ and -1 ps, is presented in Fig. S6; it clearly decreases upon increasing the K deposition time, indicating a reduction of the PV effect for lager initial band bending potentials. To understand this result, we consider the ratio of the photo-charges responsible for the PV and the initial electron population in the QWSs. As chemical gating increases, the QWS's electron occupation and density of states increase, while the number of photo-charges giving rise to the PV effect stays constant. Thus, the photo-charges can be accommodated with a smaller binding energy shift, such that at the highest doping QWS1 shows only an infinitesimal energy shift.

The Rashba spin-orbit coupling – a direct consequence of the change in the electric field in the SCR – will scale in the same way. Hence, the optical manipulation of 2DEGs will have a larger impact the smaller the surface gating.

---

[1] Tanaka, S.-i. Utility and constraint on the use of pump-probe photoelectron spectroscopy for detecting time-resolved surface photovoltage. *Journal of Electron Spectroscopy and Related Phenomena* **185**, 152–158 (2012).

[2] Yang, S.-L., Sobota, J. A., Kirchmann, P. S. & Shen, Z.-X. Electron propagation from a photo-excited surface: implications for time-resolved photoemission. *Applied Physics A* **116**, 85–90 (2014).

[3] Ciocys, S., Morimoto, T., Moore, J. E. & Lanzara, A. Tracking surface photovoltage dipole geometry in Bi_2Se_3 with time-resolved photoemission. *Journal of Statistical Mechanics: Theory and Experiment* **2019**, 104008 (2019).

[4] Reber, T. J. *et al.* The origin and non-quasiparticle nature of Fermi arcs in $Bi_2Sr_2CaCu_2O_{8+\delta}$. *Nature Physics* **8**, 606–610 (2012).

[5] Ulstrup, S., Johannsen, J. C., Grioni, M. & Hofmann, P. Extracting the temperature of hot carriers in time- and angle-resolved photoemission. *Review of Scientific Instruments* **85**, 013907 (2014).

[6] King, P. D. C., Veal, T. D. & McConville, C. F. Nonparabolic coupled Poisson-Schrödinger solutions for quantized electron accumulation layers: Band bending, charge profile, and subbands at InN surfaces. *Physical Review B* **77**, 125305 (2008).

[7] Michiardi, M. P-MTFA-S band bending simulation (2021). URL `https://github.com/okio-mm/P-MTFA-S_band_bending_simulation`.

[8] Gao, Y.-B., He, B., Parker, D., Androulakis, I. & Heremans, J. P. Experimental study of the valence band of Bi_2Se_3. *Physical Review B* **90**, 125204 (2014).

[9] Analytis, J. G. *et al.* Bulk Fermi surface coexistence with Dirac surface state in Bi_2Se_3: A comparison of photoemission and Shubnikov–de Haas measurements. *Physical Review B* **81**, 205407 (2010).

[10] Bianchi, M. *et al.* Coexistence of the topological state and a two-dimensional electron gas on the surface of Bi_2Se_3. *Nature Communications* **1**, 128 (2010).

[11] Martinez, G. *et al.* Determination of the energy band gap of Bi_2Se_3. *Scientific Reports* **7**, 6891 (2017).

[12] Nechaev, I. A. *et al.* Evidence for a direct band gap in the topological insulator Bi_2Se_3 from theory and experiment. *Physical Review B* **87**, 121111 (2013).

[13] Silva, E. A. d. A. e., Rocca, G. C. L. & Bassani, F. Spin-split subbands and magneto-oscillations in III-V asymmetric heterostructures. *Physical Review B* **50**, 8523–8533 (1994).

[14] Kronik, L. & Shapira, Y. Surface photovoltage phenomena: theory, experiment, and applications. *Surface Science Reports* **37**, 1–206 (1999).

[15] Mora-Seró, I., Dittrich, T., Garcia-Belmonte, G. & Bisquert, J. Determination of spatial charge separation of diffusing electrons by transient photovoltage measurements. *Journal of Applied Physics* **100**, 103705 (2006).

[16] Jang, S.-L. On the theory of the surface photovoltage technique based on the flat quasi-Fermi level approximation. *Solid-State Electronics* **34**, 373–377 (1991).
